# Prospective evaluation of skin toxicities in patients receiving post-mastectomy irradiation of chest wall, supra/infraclavicular and internal mammary nodes delivered by conventional versus intensity-modulated radiotherapy technique

**DOI:** 10.18632/oncotarget.20820

**Published:** 2017-09-11

**Authors:** Juan Li, Xiaofang Wang, Jinli Ma, Xiaoli Yu, Xiaomao Guo, Zhen Zhang

**Affiliations:** ^1^ Department of Radiation Oncology, Fudan University Shanghai Cancer Center, Shanghai, China; ^2^ Department of Oncology, Shanghai Medical College, Fudan University, Shanghai, China

**Keywords:** breast cancer, post-mastectomy radiation therapy, intensity modulation, conventional technique, skin toxicity

## Abstract

**Background:**

To determine whether IMRT could decrease skin toxicities in patients undergoing PMRT of chest wall, supra/infraclavicular (SCV), and internal mammary nodes (IMN) as compared to conventional technique.

**Materials and Methods:**

Between 2009 and 2013, 106 patients treated with IMRT and 138 treated with conventional technique were followed up regularly. The skin toxicities were graded according to the CTCAE v4.0 issued by the NCI, and compared between groups.

**Results:**

Grade 3 radiation dermatitis occurred in 49 patients (35.5%) in the conventional group and 14 (13.2%) in the IMRT group, and the difference was statistically significant (*p* < 0.001), favoring IMRT. Moist desquamation at the area associated with adjacent fields’ junctions or overlaps was observed in 35 patients (71.4%) in the conventional group and none in the IMRT group (*p* = 0.023). Grade 2 telangiectasia occurred in 32 patients (23.1%) in the conventional group and 9 (8.5%) in the IMRT group; this difference was statistically significant (*p* = 0.002), in favor of IMRT as well. Telangiectasias at the sub-sites associated with adjacent fields’ junctions or overlaps were observed in 26 patients (81.2%) in the conventional group and none in the IMRT group (*p* < 0.001). Further, 21 in the conventional group, who had initial moist desquamation at the sub-sites associated with adjacent fields’ overlaps or junctions, subsequently developed skin telangiectasias at the identical sub-sites.

**Conclusions:**

IMRT-based post-mastectomy irradiation of chest wall, SCV and IMN might decrease the occurrence of initial moist desquamation as well as subsequent telangiectasia at the subsites associated with adjacent fields’ junctions or overlaps as compared to conventional technique.

## INTRODUCTION

The role of irradiation of chest wall and regional nodes is widely recognized in the management of breast cancer patients with positive axillary lymph nodes after mastectomy [1–3]. Conventional post-mastectomy radiation therapy (PMRT) is often delivered with traditional field borders. One major concern with this technique is that there exists overdose to a strip of overlapping region between medial tangential field for chest wall and separate anterior field for internal mammary nodes (IMNs) in patients indicated for IMN irradiation, which might cause significant skin injuries (e.g., radiation dermatitis and telangiectasia) at this region. Dosimetric analyses have shown that computed tomography (CT)-based inverse intensity-modulation radiotherapy (IMRT) planning to treat chest wall and nodal regions as a whole planning target volume (PTV) could improve PTV coverage and sparing of nearby structures [4, 5]. Our preliminary data demonstrated that hot spots and fields’ junction issues associated with separate fields for regional nodes could be eliminated by intensity modulation, and the inclusion of IMN in PTV did not compromise the target coverage and dose homogeneity [6]. It would be of great interest to know whether such a dosimetric superiority of IMRT-based PMRT technique could transfer into clinical advantages compared to conventional technique, in terms of reduced toxicities. We herein conduct this non-randomized prospective study to compare skin toxicities in patients undergoing PMRT with IMNs included in the total loco-regional PTV using IMRT technique versus conventional technique.

## RESULTS

### Patient, tumor and treatment characteristics

The details of patient, tumor and treatment characteristics for both groups are shown in Table 1. The median age was 51 years (range 28–70 years) in the IMRT group, and 52 years (range 26–73 years) in the conventional group. The patients in both groups had similar menopausal status, tumor locations, histology, T stages, and immunohistochemical bio-markers, e.g., ER/PR status; however, a greater percentage of patients had higher N stages in the IMRT group. The overall percentages of patients, who underwent chemotherapy, hormonal therapy, or the use of trastuzumab, did not differ significantly between the two groups.

**Table 1:** Patient, tumor and treatment characteristics

Characteristics	*N* (%)		*P* value
	IMRT group (*n =* 106)	Conventional group (*n =* 138)	
Median age (range) (years)	51 (28–70)	52 (26–73)	0.95
Menopausal status			0.52
Premenopausal	56 (52.8)	66 (47.8)	
Postmenopausal	50 (47.2)	71 (51.4)	
Perimenopausal	0	1 (0.8)	
Side			0.81
Left breast	56 (52.8)	75 (54.3)	
Right breast	50 (47.2)	63 (45.7)	
Histology			0.63
IDC	102 (96.2)	133 (96.4)	
ILC	2 (1.9)	4 (2.9)	
Other	2 (1.9)	1 (0.7)	
Tumor grade			0.81
Grade I	8 (7.5)	11 (8.0)	
Grade II	40 (37.7)	51 (36.9)	
Grade III	54 (50.9)	67 (48.6)	
Unknown	4 (3.8)	9 (6.5)	
T stage			0.50
pTx-1	35 (33.0)	46 (33.3)	
pT2	56 (52.8)	79 (57.2)	
pT3–4	15 (14.2)	13 (9.4)	
N stage			0.004
pN0	8 (7.5)	14 (10.1)	
pN1	38 (35.8)	65 (47.1)	
pN2	33 (31.1)	47 (34.1)	
pN3	27 (25.5)	12 (8.7)	
Median No. of nodes removed (range)	18 (9–39)	19 (7–43)	
ER/PR status			0.18
Positive	73 (68.9)	107 (77.5)	
Negative	33 (31.1)	30 (21.7)	
Unknown	0	1 (0.8)	
CerbB2 status			0.31
−/+	72 (67.9)	104 (75.4)	
++	8 (7.5)	11 (8.0)	
+++	26 (24.5)	23 (16.6)	
Neoadjuvant chemotherapy			0.06
Yes	31 (29.2)	26 (18.8)	
No	75 (70.8)	112 (81.2)	
Adjuvant chemotherapy			0.21
Yes	86 (81.1)	120 (87.0)	
No	20 (18.9)	18 (13.0)	
Use of trastuzumab			0.07
Yes	22 (20.8)	17 (12.3)	
No	84 (79.2)	121 (87.7)	
Hormonal therapy			0.18
Yes	70 (66.0)	102 (73.9)	
No	36 (34.0)	36 (26.1)	

Abbreviations: IDC = invasive ductal carcinoma; ILC = invasive lobular carcinoma; ER = estrogen receptor; PR = progesterone receptor; IMN = internal mammary nodes; IMRT = intensity modulated radiation therapy.

### Radiation dermatitis

Overall, 31 patients (29.2%) in the IMRT group and 50 patients (36.2%) in the conventional group were identified to have Grade 2 radiation dermatitis (*X*^2^ = 1.31, *p* = 0.25). Of these, 31 patients in the IMRT group and 47 in the conventional group developed patchy moist desquamation in the axillary area; the others had moderate to brisk erythema in the chest wall. The rates of Grade 3 radiation dermatitis, i.e., moist desquamation in chest wall, other than anterior axillary skin folds and creases, were 13.2% (14/106) in the IMRT group and 35.5% (49/138) in the conventional group, respectively; This difference in the rates of Grade 3 radiation dermatitis was statistically significant between groups (*X*^2^ = 12.78, *p* < 0.001), favoring IMRT technique. The median time to the onset of moist desquamation was 6 (4–7) weeks from start of RT for both groups.

Table 2 shows the distribution of moist desquamation by occurrence sites and treatment groups. All moist desquamations occurred where bolus was applied. As can be seen, the moist desquamation is more frequently identified in the axillary area than in chest wall, which is consistent in both groups; however, a greater percentage of patients in the conventional group developed moist desquamation in chest wall (*X*^2^ = 7.2, *p* = 0.027).

**Table 2:** The distribution of moist desquamation occurrence areas

Site of moist desquamation	*N* (%)		*X*^2^	*P* value
	IMRT group (*n =* 45)	Conventional group (*n =* 96)		
Axillary area only	31 (65.9%)	47 (49.0%)	7.2	0.027
Chest wall only	5 (10.6%)	30 (31.2%)		
Both	9 (23.5%)	19 (19.8%)		

Abbreviations: IDC = invasive ductal carcinoma; ILC = invasive lobular carcinoma; ER = estrogen receptor; PR = progesterone receptor; IMN = internal mammary nodes; IMRT = intensity modulated radiation therapy.

**Figure 1:**
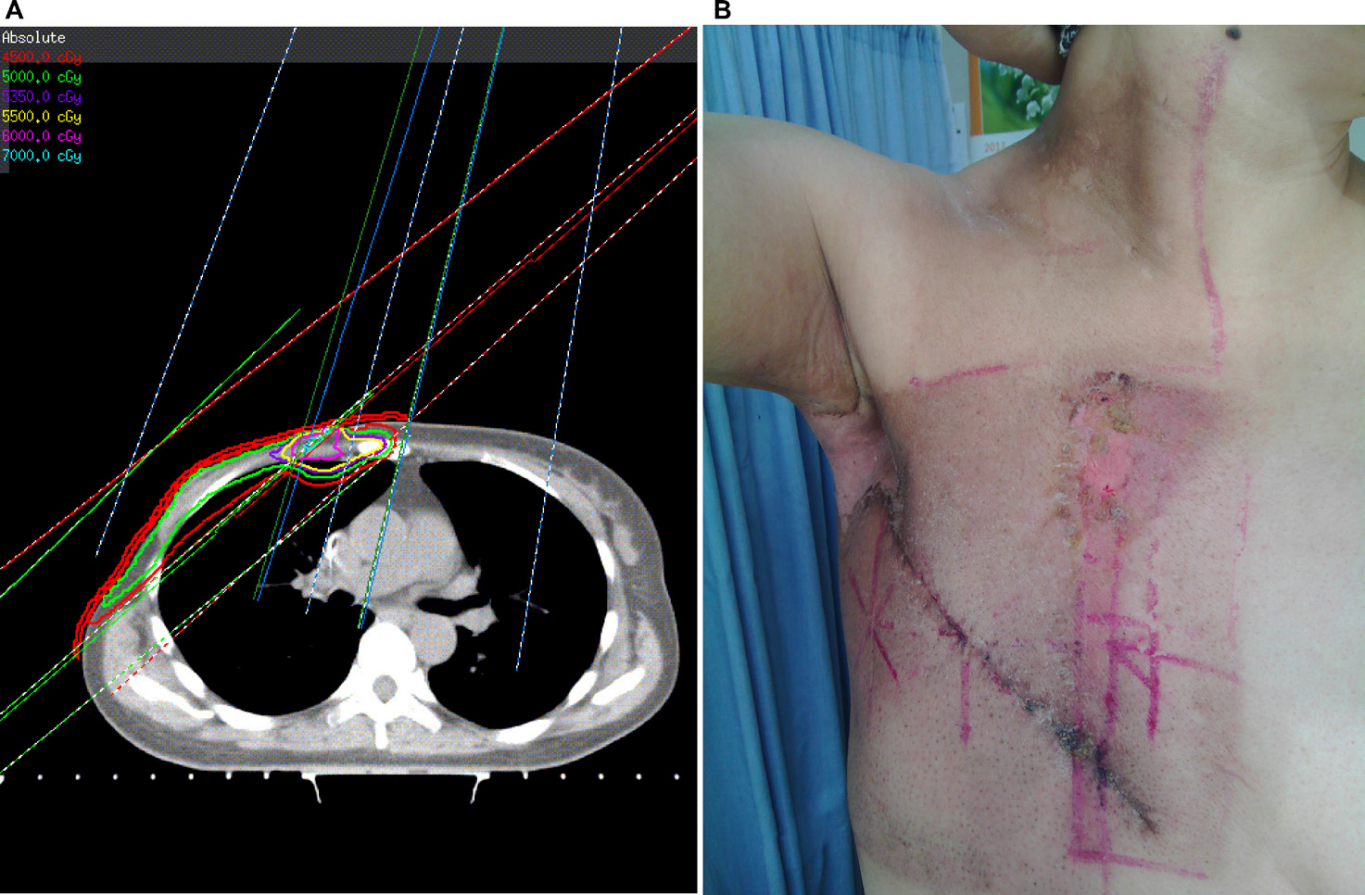
A typical post-mastectomy case with right breast cancer treated with conventional technique (**A**) a strip of skin with hot spots existed in the overlapping region between medial tangent of chest wall field and IMN field (red line: 45 Gy; green line: 50 Gy; blue line: 53.5 Gy; yellow line: 55 Gy; pink line: 60 Gy; bright blue line: 70 Gy); (**B**) moist desquamation developed in both axilla and aforementioned overlapping region of chest wall.

Table 3 compares the percentage of patients with moist desquamation at sub-sites of chest wall in the two groups. Out of 49 patients in the conventional group, 32 (65.3%) were observed to develop moist desquamation in the sterna/parasternal area, basically the overlapping region between medial tangent of chest wall field and IMN field, 10 (20.4%) at the SCV and chest wall fields’ junctions, 18 (36.7%) around the surgical scars, and 9 (18.4%) distributing elsewhere of chest wall. However, among 14 patients in the IMRT group, 9 (64.3%) were observed to have moist desquamation around surgical scars, and 8 (57.1%) developed moist desquamation elsewhere of chest wall. Overall, there were 35 (71.4%) patients in the conventional group and none in the IMRT group developing moist desquamation at the sub-sites associated with adjacent fields’ junctions or overlaps; the difference in the sub-site distribution of moist desquamation between groups was statistically significant (*X*^*2*^ = 5.1, *p* = 0.023), favoring IMRT technique. Figure 1 shows a typical case who was identified to have moist desquamation in both the axillary area and parasternal area following PMRT with conventional technique.

**Table 3:** The distribution of moist desquamation in sub-sites of chest wall by treatment group

Sub-sites of moist desquamation	*N* (%)	
	IMRT group (*n =* 14)	Conventional group (*n =* 49)
Overlapping area between medial tangent and IMN field only (a)	NA	19 (38.8%)
SCV and chest wall fields’ junctions only (b)	NA	3 (6.1%)
Around surgical scars only (c)	6 (42.9%)	8 (16.3%)
Elsewhere only (d)	5 (35.7%)	2 (4.1%)
(a) and (b)	NA	4 (8.2%)
(c) and (d)	3 (21.4%)	4 (8.2%)
(a) and (c)	0	6 (12.2%)
(a), (b), and (d)	0	3 (6.1%)

Abbreviations: NA = not applicable; IDC = invasive ductal carcinoma; ILC = invasive lobular carcinoma; ER = estrogen receptor; PR = progesterone receptor; IMN = internal mammary nodes; IMRT = intensity modulated radiation therapy.

### Radiation telangiectasia

Overall, 9 patients (8.5%) in the IMRT group and 32 (23.1%) in the conventional group were observed to have moderate number of telangiectasia (Grade 2); this difference in the incidence of Grade 2 telangiectasia between groups was statistically significant (*X^2^* = 9.2, *p* = 0.002).

All telangiectasias were located in the irradiated chest wall. Table 4 compares the percentage of patients with telangiectasia at sub-sites of chest wall in the two groups. In the conventional group, the parasternal area, SCV/chest wall fields’ junctions, the area around surgical scars, and elsewhere were involved in 78.1% (*n* = 25), 18.8% (*n* = 6), 34.4% (*n* = 11), and 9.4% (*n* = 3) of patients, respectively; whereas in the IMRT group, the area around surgical scars, and elsewhere of chest wall were involved in 66.7% (*n* = 6) and 44.4% (*n* = 4) of patients, respectively. Overall, there were 26 (81.2%) patients in the conventional group and no one in the IMRT group developing telangiectasias at the sub-sites associated with adjacent fields’ junctions or overlaps; the difference in the distribution of telangiectasia at specific sub-sites of chest wall was statistically significant (*X*^*2*^ = 19.9, *p* < 0.001), in favor of IMRT technique.

**Table 4:** The distribution of telangiectasia in sub-sites of chest wall by treatment group

Sub-sites of moist telangiectasia	*N* (%)	
	IMRT group (*n =* 9)	Conventional group (*n =* 32)
Overlapping area between medial tangent and IMN field only (a)	NA	15 (46.9%)
SCV and chest wall fields’ junctions only (b)	NA	1 (3.1%)
Around surgical scars only (c)	5 (55.6%)	4 (12.5%)
Elsewhere only (d)	3 (33.3%)	0
(a) and (b)	NA	4 (12.5%)
(c) and (d)	1 (11.1%)	2 (6.3%)
(a) and (c)	NA	5 (15.6%)
(a), (b), and (d)	NA	1 (3.1%)

Abbreviations: NA = not applicable; IDC = invasive ductal carcinoma; ILC = invasive lobular carcinoma; ER = estrogen receptor; PR = progesterone receptor; IMN = internal mammary nodes; IMRT = intensity modulated radiation therapy.

**Figure 2:**
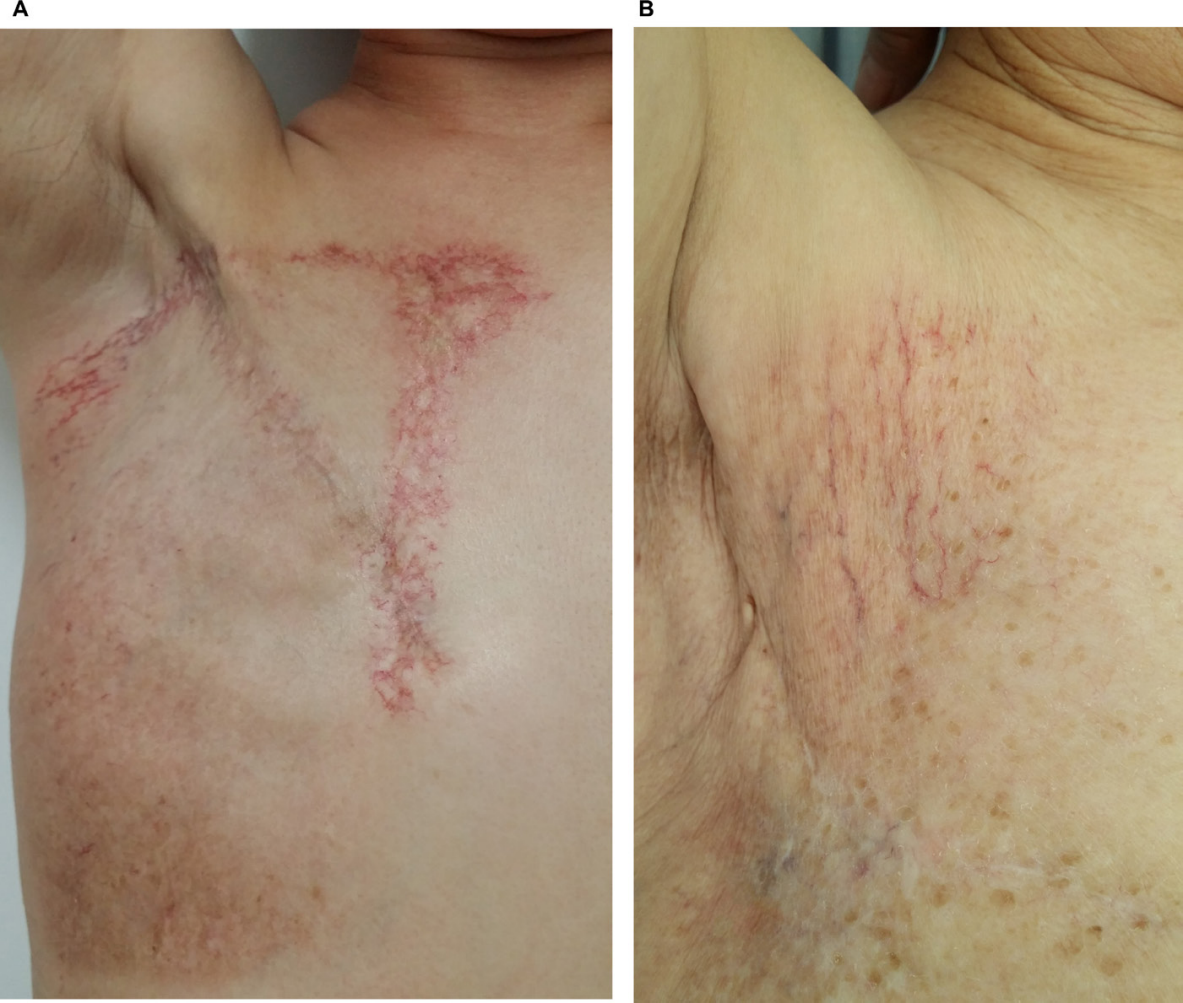
(**A**) A typical case with right breast cancer who developed skin telangiectasia at the following 3 sub-sites after PMRT with conventional technique. The overlapping region between medial tangent and IMN fields, SCV/chest wall fields’ junctions and elsewhere of chest wall; (**B**) a typical case with right breast cancer whose skin telangiectasia occurred elsewhere of chest wall following IMRT-based PMRT.

Further analysis showed that 25 (78.1%) patients in the conventional group and 3 (33.3%) patients in the IMRT group developed skin telangiectasias at the identical sub-sites of chest wall where previous moist desquamation occurred. Of those, 21 patients in the conventional group initially had moist desquamations at the sub-sites associated with adjacent fields’ overlaps or junctions, and subsequently developed skin telangiectasias at these identical sub-sites. Figure 2A shows a case who developed skin telangiectasias at the following 3 sub-sites after PMRT with conventional technique: the overlapping region between medial tangent and IMN fields, SCV/chest wall fields’ junction and elsewhere of chest wall, and Figure 2B shows a case whose skin telangiectasias occurred elsewhere of chest wall following IMRT-based PMRT.

## DISCUSSION

PMRT is an integral component of the curative treatment of breast cancer that contributes to both improved locoregional control and overall survival in appropriately selected cases. The conventional PMRT technique utilized in patients with indication of IMN irradiation generally includes a separate anterior field for IMNs with mixed photon-electron beams, except for two opposed tangential chest wall photon fields and an anterior SCV mixed beams field. The disadvantages of this technique, such as inhomogeneous dose distribution associated with the use of mixed beams for regional nodes, under or overdose because of the adjacent field junctions or overlaps, and suboptimal coverage of regional nodes, have been discussed in detail previously [6]. Thus far, many ways have been explored to address field junction issues and improve dose distribution [7]. Among these, CT-based multi-field IMRT planning to treat chest wall and regional nodes as a whole PTV has been proved to be dosimetrically feasible and clinically well-tolerated by most patients [6]. Unfortunately, to the best of our knowledge, no prospective studies have as yet been initiated to compare the toxicity profile following post-mastectomy irradiation of chest wall and regional nodes with conventional technique versus IMRT technique.

In the present study, post-mastectomy breast cancer patients indicated for IMN irradiation were prospectively treated with inverse IMRT technique versus conventional technique by the same set of clinicians during the same time period. The skin toxicities commonly seen after PMRT, such as radiation dermatitis and telangiectasia, were evaluated and compared between groups.

### Radiation dermatitis

As with previous reports, radiation dermatitis is demonstrated to be the most common acute side effect in this study, regardless of the PMRT technique used. The frequency of ≥ Grade 2 radiation dermatitis was 29.2% for IMRT group, as compared to 36.2% for conventional group. This high rate is not unexpected and is compatible with prior studies [8]. Though a greater number of patients who received conventional PMRT were observed to develop ≥ Grade 2 radiation dermatitis, the difference was non-statistically significant. However, further analysis showed a statistically significant trend for a greater incidence of Grade 3 radiation dermatitis in the conventional group compared with patients treated with IMRT technique (32.3% vs. 12.9%, *p* value < 0.001).

The moist desquamation tends to occur at areas with skin folds or creases, and easy to sweat, especially where bolus was applied and a hot spot of skin dose located. The most common site for moist desquamation is the axillary area in this study, which is consistent with previous reports [9] and does not depend on the PMRT technique used. It’s not difficult to understand because the axillary area is full of sweat glands and skin creases. Nonetheless, moist desquamation over the chest wall is more frequently seen in patients treated with conventional PMRT technique. In other words, fewer patients in our IMRT-based PMRT series suffered chest wall moist desquamation, which might be attributed to the improved homogeneity of dose distribution in chest wall by intensity modulation. As we have reported, the percent volume of PTV receiving ≥ 110% of prescription dose was negligible in the IMRT-based PMRT series, whereas in the conventional PMRT series, there always existed a strip of skin with hot spots in the overlapping region between medial tangent of chest wall field and IMN field [6]. Thus, hot spots, fields’ junction and overlap issues associated with separate fields for regional nodes were basically eliminated by intensity modulation. Patients requiring IMN irradiation therefore most likely benefit from this IMRT-based PMRT technique, in terms of acute skin toxicities.

### Radiation-induced telangiectasia

Radiation-induced telangiectasia is a well-recognized manifestation of late cutaneous radiation damage after radiation therapy for breast cancer. It’s usually confined to the site of highest dose, and can be very unsightly depending on its site, size and extent. It usually becomes apparent at 1–2 years after completion of treatment, and may slowly progress over subsequent years. In some cases, it is the most obvious reminder to the patient of their illness and its treatment [10, 11]. It’s challenging to clinically prevent or manage such a distressing condition without compromising tumor control.

In addition to radiation boost to the tumor bed or mastectomy scar, the hotspots of skin dose associated with conventional PMRT technique for patients with indication of IMN irradiation represent another risk for the development of skin telangiectasia. As is similar to the above-mentioned finding on moist desquamation, this prospective study demonstrated that a smaller percentage of patients in the IMRT group suffered skin telangiectasia of chest wall than in the conventional group, and almost none occurred at areas next to the sternum or around SCV/chest wall fields’ junctions. From another point of view, due to the hot spots frequently seen at the overlapping region of medial tangent and IMN fields, and sometimes at the SCV/chest wall fields’ junctions, a considerable proportion of patients in the conventional group, who had initial moist desquamations at these sub-sites, were noted to develop subsequent skin telangiectasias at the same sub-sites. Therefore, compared to conventional technique, the improved dose homogeneity by intensity modulation might reduce the occurrence of skin telangiectasia as well as moist desquamation at sub-sites of chest wall associated with adjacent fields’ junctions or overlaps.

### Limitations and future directions

This study has some limitations which need to be addressed. First, it’s necessary to be conscious of the inherent pitfalls of a prospective but non-randomized study, particularly of the potential extent of observer bias involved. Second, apart from hot spots of skin dose, several other risk factors, such as BMI, treatment time, and skin types, have been shown to be associated with the occurrence of moist desquamation. Unfortunately, patients’ data regarding BMI and skin types were not available on our medical records to assess whether there was an association between these factors and the occurrence of moist desquamation. Last, skin toxicities definitely impact a patient’s QOL. It would be of great interest to know whether IMRT-based PMRT technique could improve patients’ QOL by reducing the incidence of both early and late severe skin toxicities, as compared to conventional technique. In future, a prospective randomized clinical trial comparing conventional technique vs. IMRT for PMRT might help to answer some questions about patients’ tolerance, QOL, and local control, and finally determine whether IMRT-based PMRT to treat SCV, IMN and chest wall as a whole PTV should become new standard replacing the traditional field design.

## MATERIALS AND METHODS

### Study population

Between June 2009 and May 2013, a total of 299 patients, who signed an informed consent form approved by the local Ethics Committee, were prospectively enrolled onto this study and completed PMRT with IMNs as a part of CTV at our institute. Of these, 244 patients, who had regular follow-up information, were included in the current analysis.

### Radiotherapy techniques

According to the protocol of this study, patients were sequentially allocated into the IMRT or conventional group by a research nurse at time of registration. However, the treating physicians retained the final right to decide which technique to use for a specific patient. Generally, patients with thoracic deformity or higher nodal stages were more likely to be treated with IMRT technique. Before simulation, each patient was placed supine on a commercially available breast tilt board (Med-Tech 350) to make sternum parallel to the table, with both arms fully abducted (90 degrees or greater) and externally rotated, and head position secured. All treatments were delivered with 6-MV photon using an Electa linear accelerator. To ensure a sufficient skin dose, a daily 3-mm bolus was placed on chest wall of each patient.

### IMRT group (*n* = 106)

Generally, a planning CT scan at 5-mm intervals from mid-neck to diaphragm was obtained for each patient in the treatment position using an AcQsim CT simulator (Philips Medical Systems). At CT simulation, the mastectomy scar was routinely wired with radiopaque markers. The CTVs including ipsilateral chest wall, mastectomy scar, supra/infraclavicular region, and IMN were contoured, and expanded to form a whole PTV. The anatomic borders of these CTVs and corresponding PTV expansions have been described in detail previously [6]. For each patient, a multi-beam IMRT plan was designed using either integrated full beams or two segments of half beams split at caudal edge of clavicle head. The prescription dose was 50 Gy in 25 fractions. All plans were optimized to cover the whole PTVs (90% of PTV to receive 50 Gy), and spare surrounding normal tissues (ipsilateral mean lung dose ≤ 20 Gy, and ≤ 30% of the ipsilateral lung to receive ≤ 20 Gy; ≤ 5% of the heart to receive ≤ 30 Gy, mean heart dose ≤ 10 Gy for left-sided lesions; spinal cord maximum dose ≤ 45 Gy; contralateral breast mean dose ≤ 1.5 Gy) as much as possible. After optimization, plan evaluation was performed for each patient to count for hot spots of skin dose. The percent volume of PTV receiving ≥ 110% of prescription dose was negligible (< 5%) and scattered in whole PTV.

### Conventional group (*n* = 138)

Conventional technique includes tangential chest wall fields, a separate anterior medial oblique supraclavicular field (angled approximately 10 or 15 degree to the contralateral side, i.e., medial-anterior oblique), and a separate anterior or anterior-oblique IMN field. CT scans were acquired to optimize RT plans after field borders were determined at conventional simulation. Generally, tangential chest wall plans were optimized with field-in-field technique using 6-MV photon to achieve desired dose uniformity. Supraclavicular and IMN fields were also planned on CT scans using mixed electron-photon beams. All sites were to be treated to 50 Gy at 2 Gy per fraction. The maximum dose in the overlapping region between medial tangent and IMN fields was confined to below 75 Gy and the volume receiving > 110% prescription dose was minimized. However, all patients in the conventional group were found to receive overdose to a strip of the overlapping region at time of plan evaluation.

### Adjuvant systemic therapy

The indications to offer systemic therapy were not fixed in this study. The actual systemic therapies were generally given according to international guidelines. Basically, most patients were given chemotherapy, ER/PR positive patients were given hormonal therapy, and Her-2 positive patients were recommended to use one year of trastuzumab.

### Assessment of skin toxicities

All patients were clinically inspected by an experienced investigator before, during and after radiation therapy. Radiation dermatitis was checked weekly from start of RT until 4 weeks after RT, and telangiectasia was checked every 6 months for at least 2 years after the completion of treatment. At time of each visit, clinical findings were recorded, and pictures of the irradiated area were taken by the research nurse to document skin toxicity. Based on the clinical inspection and pictures, all skin toxicities were graded by the experienced investigator according to the common terminology of criteria for adverse events version 4.0 (CTCAE v4.0) issued by the National Cancer Institute. The radiation dermatitis and telangiectasia of maximum grade were recorded for each patient. In order to conveniently document the location of maximum grade skin toxicities, chest wall were divided into the following 4 sub-sites: (a) sternal/parasternal area, which included the overlapping region between medial tangent and IMN field, (b) area associated with SCV/chest wall fields’ junctions, (c) area around surgical scars, and (d) elsewhere.

### Statistical analysis

Differences in the rates and sub-site distribution of skin toxicities between treatment groups were compared using Chi-square test. *p* values of ≤ 0.05 were considered statistically significant.

## CONCLUSIONS

This prospective study revealed a lower incidence of both radiation dermatitis and telangiectasia of chest wall in patients receiving IMRT-based post-mastectomy irradiation of chest wall, SCV, and IMN. The elimination of hot spots of skin dose at the overlapping region of IMN and medial tangent of chest wall fields by intensity modulation might decrease initial moist desquamation as well as subsequent telangiectasia at this sub-site, and IMRT-based PMRT technique therefore most likely benefit those patients indicated for IMN irradiation.

## References

[1] Ragaz J, Olivotto IA, Spinelli JJ, Phillips N, Jackson SM, Wilson KS, Knowling MA, Coppin CM, Weir L, Gelmon K, Le N, Durand R, Coldman AJ (2005). Locoregional radiation therapy in patients with high-risk breast cancer receiving adjuvant chemotherapy: 20-year results of the British Columbia randomized trial. J Natl Cancer Inst.

[2] Verma V, Vicini F, Tendulkar RD, Khan AJ, Wobb J, Edwards-Bennett S, Desai A, Shah C (2016). Role of Internal Mammary Node Radiation as a Part of Modern Breast Cancer Radiation Therapy: A Systematic Review. Int J Radiat Oncol Biol Phys.

[3] Recht A, Comen EA, Fine RE, Fleming GF, Hardenbergh PH, Ho AY, Hudis CA, Hwang ES, Kirshner JJ, Morrow M, Salerno KE, Sledge GW (2016). Postmastectomy Radiotherapy: An American Society of Clinical Oncology, American Society for Radiation Oncology, and Society of Surgical Oncology Focused Guideline Update. Pract Radiat Oncol.

[4] Rudat V, Alaradi AA, Mohamed A, Ai-Yahya K, Altuwaijri S (2011). Tangential beam IMRT versus tangential beam 3D-CRT of the chest wall in postmastectomy breast cancer patients: a dosimetric comparison. Radiat Oncol.

[5] Jagsi R, Moran J, Marsh R, Masi K, Griffith KA, Pierce LJ (2010). Evaluation of four techniques using intensity-modulated radiation therapy for comprehensive locoregional irradiation of breast cancer. Int J Radiat Oncol Biol Phys.

[6] Ma J, Li J, Xie J, Chen J, Zhu C, Cai G, Zhang Z, Guo X (2013). Post mastectomy linac IMRT irradiation of chest wall and regional nodes: dosimetry data and acute toxicities. Radiat Oncol.

[7] Klein EE, Taylor M, Michaletz-Lorenz M, Zoeller D, Umfleet W (1994). A mono isocentric technique for breast and regional nodal therapy using dual asymmetric jaws. Int J Radiat Oncol Biol Phys.

[8] Bostrom A, Lindman H, Swartling C, Berne B, Bergh J (2001). Potent corticosteroid cream (mometasone furoate) significantly reduces acute radiation dermatitis: results from a double-blind, randomized study. Radiother Oncol.

[9] Hymes SR, Strom EA, Fife C (2006). Radiation dermatitis: clinical presentation, pathophysiology, and treatment 2006. J Am Acad Dermatol.

[10] Rowland Payne CM, Somaiah N, Neal AJ, Glees JP (2005). The hyfrecator: a treatment for radiation induced telangiectasia in breast cancer patients. Br J Radiol.

[11] Fehlauer F, Tribius S, Holler U, Rades D, Kuhlmey A, Bajrovic A, Alberti W (2003). Long-term radiation sequelae after breast-conserving therapy in women with early-stage breast cancer: an observational study using the LENT-SOMA scoring system. Int J Radiat Oncol Biol Phys.

